# Leiomyoma as a case of renal tumor

**DOI:** 10.1016/j.eucr.2021.101792

**Published:** 2021-07-29

**Authors:** Piotr Kamiński, Adam Nogalski, Dorota Lewkowicz, Janusz Ciechan

**Affiliations:** a Department of Traumatology and Emergency Medicine, Medical University of Lublin, Staszica 21, 20-081, Lublin, Poland; b Department of Clinical Pathomorphology, Medical University of Lublin, Jaczewskiego 8b, 20-090, Lublin, Poland; c Department of Urology, Regional Specialist Hospital, Bema 1, 24-100, Puławy, Poland

**Keywords:** Renal tumor, Leiomyoma, Radical nephrectomy

## Abstract

Leiomyoma is a benign tumor originate from mesenchymal or connective tissue. Because of a low incidence, difficulties in differentiation from other renal tumors with imaging modalities, the definitive diagnosis of a leiomyoma is possible after examination of a specimen. We present a case of 79-years-old women with incidentally discovered renal tumor in CT scan. Because of the small size of a tumor, patient was informed about possibility of active surveillance. Partial nephrectomy was performed with a histopathologic diagnosis of renal leiomyoma. After 6 months of a follow-up, patient is found to be asymptomatic and free of disease.

## 1 Introduction

Renal tumors are frequently diagnosed urological neoplasms and renal cell carcinoma (RCC) represents 90% of them. Leiomyoma is an extremely rare CASE of a tumor arising from smooth muscle or connective tissue. Till today, differentiation diagnosis of renal masses with imaging modalities is not feasible. The definitive diagnosis is only possible after radical excision of a tumor followed by pathologic examination. We report a case of 79-year-old women with incidentally found renal mass treated with partial nephrectomy.

## 2 Case presentation

**Fig. 1:**
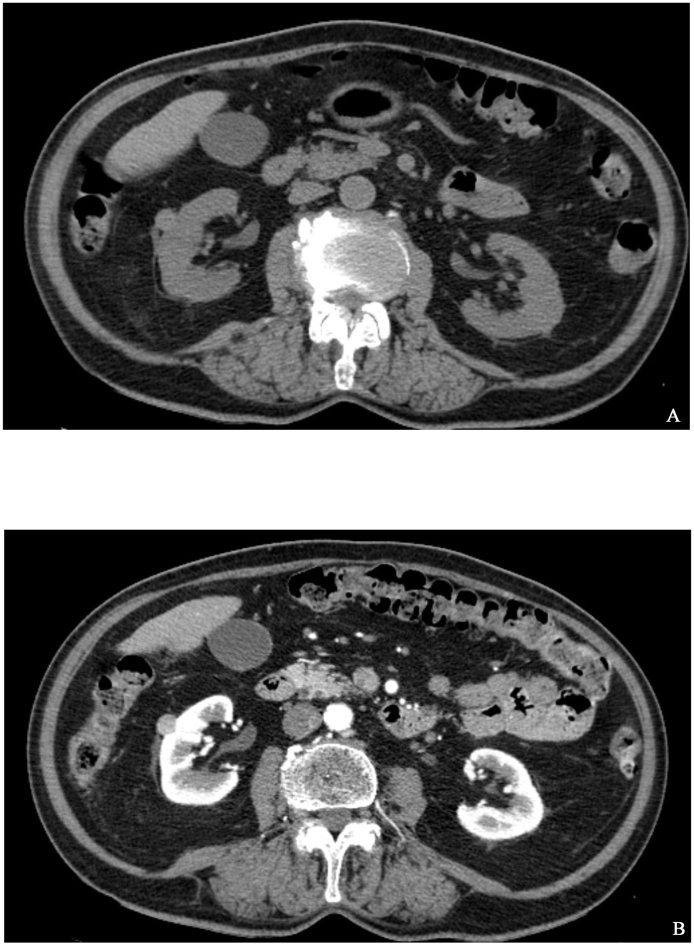
Abdominal CT scans. A. Non-enhanced CT shows subcapsular mass in right kidney; B.) CT with contrast, arterial phase, shows well-circumscribed, high-density solid lesion with enhancement.

A 79-year-old female was administered to urology department because of a renal tumor, incidentally found in CT scan. Before that, patient was diagnosed in general surgery clinic because of unspecified abdominal pain and a weight loss. Abdominal US was performed and the pathological mass in pancreas was found. CT scan excluded pancreatic tumor but detected 12 mm diameter tumor of the central part of right kidney, described as a subcapsular, well-circumscribed, high-density solid lesion with contrast enhancement [Fig. 1]. Serum creatine was normal and there was no microscopic hematuria in urinalysis. With clinical diagnosis of small RCC, patient was informed about active surveillance (AS) protocol and other therapeutic options and chose surgical treatment. Partial nephrectomy was performed, post operative period was uneventful. On gross examination of the specimen, 15mm, well-circumscribed tumor was found, surgical margins were clear. Immunohistochemical markers were performed and expression of SMA and desmin was detected but s-100 and CD-34 markers had no expression. The final diagnosis of leiomyoma was made. After 6 months of a follow-up, patient is found to be asymptomatic and free of disease.

## 3 Discussion

Leiomyoma is uncommon, benign renal tumor, originate from mesenchymal or connective tissue. Usually, it is more common in uterus or gastrointestinal tract. According to Romero at all the incidence is 0,3% of all surgically treated patients with renal lesions whereas data from autopsy studies suggests that it occurs in 4–5,5%.^1^ Leiomyoma is more common in women (two-third of cases) and in the Caucasian population. Nowadays, the frequent use of imaging techniques has led to growth in incidental diagnosed renal tumors, including leiomyomas.

Symptomatic cases are mostly reported before fifth decade of age but incidentally found lesions are diagnosed in elderly patients, only few cases were described in children.^1^ Most common symptoms are palpable mass (57%), abdominal/flank pain (53%) or gross hematuria (20%).^2^ Usually tumor affects lower pole of kidney (75%), there is no predilection to left or right side. Leiomyoma arises from any area with smooth muscle, according to Steiner most commonly diagnosed in subcapsular (53%) and capsular area of kidney (10%) and in renal pelvis (10%).^3^

The differential diagnosis of small, solid renal tumor includes other benign and also malignant lesions like RCC. Unfortunately, diagnostic criteria of leiomyoma by imaging modalities has not been defined yet. Usually, on ultrasonography it appears as a solid, heterogeneous, well-defined, hypoechoic masses but also as cystic lesions. CT scans display some differences depending on the size of the tumor. Small lesions are well-circumscribed, solid or cystic masses, with homogeneous enhancement on contrast administration. Large renal masses are heterogenous after contrast administration because of cystic or hemorrhage degeneration; extension into renal pelvis or outside the kidney is possible.

**Fig. 2:**
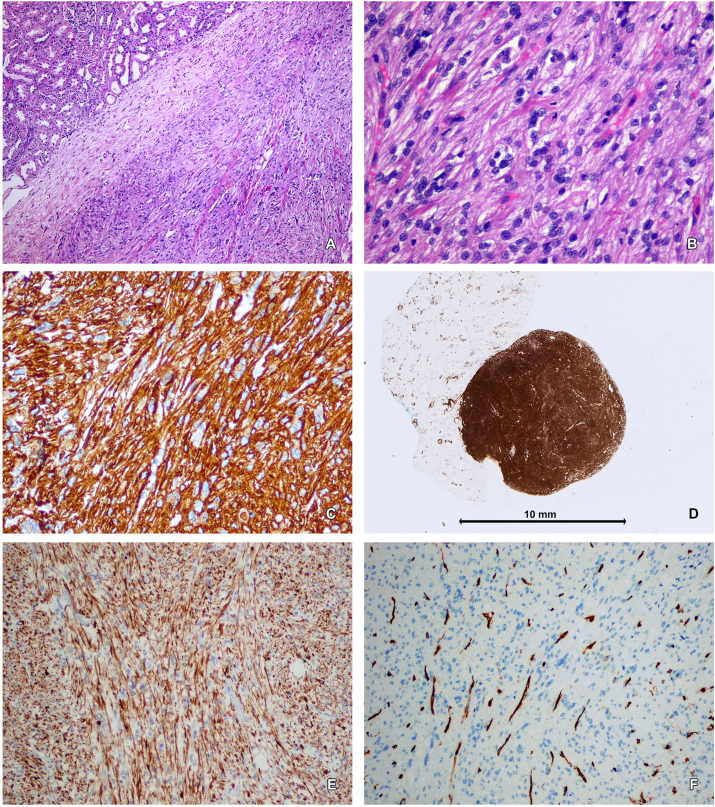
Leiomyoma of the kidney. AB. Well defined, unencapsulated tumor composed of spindle cells containing oval or cigar-shaped nuclei without atypia and mitotic figures (H + E, objective magnification A-5 × , B-20 × ); C. Strong positive immunohistochemical reaction for desmin in tumor cells (objective magnification 20 × ); DE. Positive immunostaining for smooth muscle actin (SMA) in neoplastic cells (E − objective magnification 10 × ); F. Negative immunostaining for CD34 in tumor cells – positive reaction observed in vessels walls (objective magnification 10 × ).

Finally, diagnosis of renal leiomyoma is confirmed only by pathologic examination with immunohistochemical (IHC) markers. This examination is be performed from specimen after surgical treatment or tissues from percutaneus sampling. Renal biopsy is indicated for candidates for AS, radiologically indefinite renal mass or to obtain histology before ablative treatment. In this CASE, we didn't perform biopsy because of low experience in renal biopsies and patient decision to perform a surgical treatment. Patill et colleagues described pathological diagnostic criteria of leiomyoma. On microscopy, cells have scant to moderate cytoplasm with elongated cylindrical blunt-ended “cigar-shaped” nuclei without atypia, necrosis and with low mitotic activity. On the other hand, the presence of necrosis, cytologic atypia and mitotic activity, with or without smooth muscle cells are diagnostic criteria od leiomyosarcoma. In case of diagnostic difficulties, increased proliferation assessed as Ki67(>5%) confirmed the diagnosis of leiomyosarcoma.^4^ The IHC staining results were also described in this study. In 100% of cases they observed nonfocal positive staining for desmin and negative for HMB-45 and cathepsin K. The researchers also assessed immunoreactivity of estrogen receptor (ER) end progesterone receptor (PR). Gupta and colleagues observed that 87% of cases were positive for PR and ER. In presented case, IHC staining was performed only for desmin, SMA and CD-34 but not for a ER, PR and HMB-45 [Fig. 2]. The potential role of ER and PR in development of renal leiomyoma has not been evaluated yet. Furthermore, it is noteworthy that potential impact of estrogen and progesterone in growth of gynecological leiomyoma is well-known. In case of fact, that the most of described cases occurred in postmenopausal women, raises the question of possible affect of sex steroid hormones in pathogenesis of renal leiomyoma.

Imaging modalities cannot allow to distinguish leiomyoma from RCC. However, surgical approach remains only available treatment option. Tumor size and its location are two main features of choosing between radical or partial nephrectomy. There are limited data about preferred surgical approach. Only Patill and all report nine cases of leiomyoma, in five cases nephron-sparing surgery was performed whereas 4 patients underwent radical nephrectomy.^5^

Follow-up criteria has not been defined yet. There are also limited data from long-term observational studies after radical treatment. In study conducted by Gupta and all, only in 10 cases follow-up data were available. Median follow-up was 53 months. None of the patient developed metastatic disease or died because of the tumor. 80% of them were alive without the disease and 20% of them died because of other disease.^4^

## 4 Conclusion

In conclusion, renal leiomyomas are extremely rare tumors of a kidney with predilection to a female patients. It is noteworthy that differentiation between leiomyoma from other renal lesions like RCC or others is based only on clinical factors and imaging modalities. In most cases, it is impossible to distinguish between different types of kidney tumors before histopathologic examination; surgical removal of a tumor or kidney remains only available treatment option.

## Funding

No funding.

## Declaration of competing interests

No competing interests.
